# Community-networks that facilitate engagement in health research: Ifakara Health Research Institute-Bagamoyo case study

**DOI:** 10.12688/aasopenres.13187.1

**Published:** 2021-03-11

**Authors:** Leah Bategereza, Ally Olotu, Dorcas Kamuya

**Affiliations:** 1 Ifakara Health Institute, Bagamoyo, Tanzania; 2 School of Life Science and Bio-engineering, The Nelson Mandela African Institution of Science and Technology, Arusha, Tanzania; 3 Kenya Medical Research Institute (KEMRI), Kilifi, Kenya

**Keywords:** Community networks/structures, community engagement, health research

## Abstract

**Background:** Involvement of communities in the field of health research has been at the forefront of what is considered as ethical conduct of research. A commonly used approach is regular meetings with locally recognized community leaders to consult about research activities, i.e. community engagement. At the Ifakara Health Research Institute (IHI) in Bagamoyo, Tanzania, different approaches to engaging with the community in health research have been used, but there has not been a systematic understanding of the functioning of the community network that is engaged within health research.

**Methods:** To understand the community networks engaged in health research, perceptions of community stakeholders and researchers on the functionality of the community networks was performed. We conducted six focus group discussions with respondents who have participated in IHI research for the past five years and 49 in-depth interviews.

**Results:** Community networks involved in engagement were influenced by the type of research project and kind of participants needed. Different community networks were involved in engagement activities, namely village executive officers, community health workers, hamlet leaders, nurses, doctors and community advisory boards. Approaches used during engagement processes to inform potential participants about the work of IHI and specific studies that are undertaken were useful in passing key information, however, they did not always reach the target population due to having limited levels of interaction with potential participants. Participants and researchers suggested additional ways to engage with the community.

**Conclusion:** There is a need of developing a community engagement unit that would work across projects to support engagement with the community. The unit will maintain continuous engagement with the community and conduct research to understand the relationship between communities we work in and researchers. Funding of this unit could be done through contributions from the core budget, individual’s projects or competitive grant application.

## Introduction

### What are community networks?

Community networks/structures are described as relevant structures in a community which the researchers consult for research-related activities, including seeking permission to engage the wider community about research activities, protecting the community, or informing the community about research. Different forms of community structure are described in the literature, including administrative leaders, religious leaders, community representatives and Community Advisory Board (CAB) ^1^. Engaging community structures about health research is important as they can provide insights into various issues that can inform the design and implementation of research; they can make researchers aware of community norms, of cultural sensitivities of the research and consent processes. They can also be enlighten the priority health needs of the community which might contribute to a better understanding of locally-relevant research ^1^. One approach of engaging the communities is through community representatives. In health research, a CAB, a representative body of community members that might be selected or elected by their communities or appointed by community leaders, is widely documented as one approach of engaging communities ^2^. Community representatives can work with research staff and be provided with opportunities to be involved in overall activities regarding the research, such as development of the research protocol, obtaining consent, collecting and reviewing data, access to data and sampling ^2^. Community engagement has been highly promoted in health delivery as well. 

### Role of community networks/structures in health research

Community engagement has been identified as an essential aspect when conducting research activities that require drawing people/participants from a particular community. With this reason, community structures/networks are there to facilitate the engagement processes as they act as a link between researchers and community members. Some of the key health research funders recognize the important contribution of community engagement in health research; for example, the Wellcome Trust expounds on the role of engagement in advancing health research, set a dedicated funding stream for engagement, and galvanized a call to other funders to join in spearheading engagement ^3^. They make a concerted call to other research funders to pay attention to and invest in engagement, noting that funders focus mostly on investing millions of dollars on product development, clinical training, designing and building of facilities, but have often ignored the important part of community engagement ^4^. Engagement with the community for any successful clinical research can be attained by making the community feel as part of the research team, and by allowing them to be involved in the things that are potential for attainment of the research goal. The interaction can be with general community or selected members or representatives of those communities. Guidelines in good participatory practice for a HIV prevention trial has elaborated importance of community engagement and consenting. It stated that the consenting process may be influenced by community engagement activities and these may be regarded as good research practice ^5^. Community engagement is a process whose activities may aim to inform and strengthen consent process and overall conduct of the research ^5^.

In a study on community engagement that was conducted in four countries, Thailand, India, South Africa and Canada, challenges encountered by the community stakeholders in facilitating engagement in biomedical HIV prevention trial were explored. Three themes identified from the study were illustrated as essential to community stakeholders. These included: (a) **Trial literacy** in which the community were educated about some of the key concepts of the trial such as placebo, controls and double-blinding. This followed communication challenges and trial-related misconceptions that were seen to contribute to low uptake of the trial in the community. (b) **Historical mistrust** is whereby conceptualizations of clinical trials and bio-medicine were described in the context of historical experiences with colonialism. Established community structure were likely to minimize the mistrust in the community since some community members regarded research as forms of colonialism. Some claimed that in trials, participants were being injected with diseases such as polio. There were deep-rooted concerns about study sites, research being perceived as rooted in the historical oppression, due to the fact that trials were happening only in Africa and the Africans were used as guinea pigs. (c) The importance of early **meaningful engagement with community** stakeholders was described as important in providing opportunities for community members to participate in the trial planning process, protocol formulation and to determine community perception on the social value of the research. Early engagement was thought to minimize historical mistrust as community stakeholders would feel that they were part of the research activity, and thus identified with some partial ownership and control of the research. They also viewed that community involvement would not end with the trial life, but that it would continue beyond study recruitment and result dissemination ^6^.

### Centrality of community networks/structure

Empirical research suggests the centrality of working with community structures to nurture mutual respect and trust in research, and to strengthen the science. This was highlighted in a study conducted in Coastal Kenya exploring perceptions and functions of a network of community member’s set-up specifically to consult about on-going and planned research activities, and the general understanding of research in the community. The network of community representatives at the KEMRI-Welcome Trust Program, is known as KEMRI-Community Representatives (KCR), and acts as a bridge between the community and KEMRI, through regular discussion about community concerns and issues with the Research Program, and advising studies on culturally and linguistically appropriateness of study processes and information in consent documents. KCR members are expected to consult their communities where they live as part of their daily routine activities. Engaging the community through the KCR network was said to have been beneficial to the Research Program. Generally knowledge about medical research in the community increased, rumors that had been circulating started being addressed, and there were some policies implemented as a result of feedback from the KCR, e.g. employment policies were changed so that fieldworkers and other support staff are employed from within the community where the research activities are conducted ^2^. However, there were also documented challenges with the network including KCR members negotiation for their own individual benefits (e.g. employing their own children in the Program), rather than negotiating for community benefits. Some threatened the Centre that if their demands were not met, they could fuel false rumors in the community about the research. This seems to suggest that there should be careful consideration of how community structures are set up, that clear mandate and roles are important, as well as ensuring that power and information about research is shared across different forms of community structures, not just concentrated in one.

In another study in Northern Kenya conducted by a collaborative programme between Kenya Medical Research Institute and the US Centers for Disease Control (KEMRI/CDC), village reporters were regarded as the community structure, and were consulted and actively involved in the research activities ^7^. Initially, village volunteers supported the research activities by providing study information and in recruiting potential participants into research activities. Their involvement was later formalized through the development of standard operating procedures that clearly stated their roles and renamed them as village reporters. These village reporters were viewed as the backbone of the community engagement programme at KEMRI/CDC. Their roles involved creating awareness and sensitizing people about current and planned studies, and about specific research activities and procedures. Their strength was based on the interaction and quick access to community they were members of (i.e. they were recruited from within their resident community). Being embedded within their community seemed to solidify their ability to create good working relations and exposure gained from KEMRI/CDC in terms of training and meetings strengthened their understanding about research. However, it is reported that the village reporters also faced some challenges. There were many research projects at KEMRI/CDC with different investigators; and each project reimbursed different rates to the village reporters which created tensions between the village reporters and across different study PIs. Another challenge was the increasing expectations and demands from community members to the village reporters. This includes demanding basic needs such as clothing, food, health care access, and school fees among others. Since village reporters relayed information about trials within their community, the research team found that the village reporters who felt dissatisfied about involvement in a specific trial ended up being passive or instigated different opinions that opposed the trial ^7^.

In a double-blind, randomized, placebo-controlled clinical trial that was conducted at Vanderbilt University Medical Center in USA on African-American women ^8^ use of community engagement principles and approaches were found to be essential in enhancing clinical trial recruitment and retention. The Community-Engaged Research Core (CERC), a CTSA-supported resource designed to facilitate community involvement in clinical and translational research, was consulted to provide assistance with the implementation of the clinical trial, and specifically to enhance participation of the target population, i.e. African American women. CERC’s key recommendations included: (a) convening of a community engagement studio, (b) redesigning of the recruitment advertisement, (c) simplifying the language used to explain the scope of the study, and (d) providing transportation for participants. As a result of these interventions, a comprehensive strategy to recruit, enroll, and retain participants was formulated. After implementation of the plan by the study team, enrollment increased to 78% and recruitment goals were met 16 months ahead of schedule. Participant retention and study drug adherence was 100% ^6^. From the above example it shows that community engagement is essential to the development of an effective multifaceted plan to improve recruitment of underrepresented groups in clinical trials. 

Drawing on these cases of community engagement using community structures, there are different forms of community structures but the common one includes community-recognized leaders/gatekeepers/representatives. These leaders are important in gaining permission to the community for a research to be conducted in an area. The evidence also suggests that community structure often act as bridge between the community members and research team, and that this role is important in strengthening research activities in a community, in promoting social-cultural consideration of communities in research activities, in promoting inclusion of community voices in research activities, and can contribute to meeting recruitment and retention targets within time. The evidence also suggests that community structures can wield considerable power gained in the intermediary role they play in research. Such power can be welded positively to promote mutually beneficial conduct of research in the community, and it can also be welded negatively through controlling the information (and promoting misinformation) that gets to their social network especially if the demands of the community structures are not met.

### The Ifakara Health Institute-Bagamoyo

Bagamoyo branch of the Ifakara Health Institute (IHI-Bagamoyo) was initiated in 2005 to conduct clinical research in Bagamoyo District, Tanzania. Subsequently, many ground-breaking clinical research studies have been conducted by the branch, including studies investigating Coarterm Pediatrics formulation, Phase III trial assessing the efficacy of a most advanced malaria vaccine candidate, RTS, S, and TB drug trials. All these clinical trials required the participation of the community members as participants ^9^. Since 2006, IHI-Bagamoyo Clinical research site has successfully conducted over 23 trials. At the time of conducting this project there were two ongoing clinical trial at the center. Of the 23 trials conducted so far, nine were malaria vaccine trials, six were malaria drugs trials, two were TB vaccine trials, four were TB drug trials, one was a micronutrient (Iron) supplementation trial, and three were diagnostics trials.

***The CAB at Ifakara Health Institute.*** In 2006, a Community Advisory Board (CAB) was established to work alongside Bagamoyo Research and Training Centre (BRTC) on research-related activities involving the community. Their role was to be mediators between the community and the researchers, informing researchers about community concerns with regards to their studies. They were also involved in dissemination of information about the research, and facilitation of interactions between BRTC and the community, ensuring that the community expressed their concerns, and these were addressed by the researchers. These levels of involvement were said to have enhanced community participation and follow up procedures during clinical trials, and helped in the management of rumors and crises ^9^. For example, there were rumors that the blood samples collected from the children was too much; this crisis was solved by gathering a meeting of community members, researchers and those whose children who were involved a lot in the research. In these meetings, different sizes of sample collecting tubes were displayed and the parents were asked to show the tubes used to collect blood. It was observed that the smallest bottle was the one used in collecting the blood samples. Right from the beginning, it was clarified that the roles of CAB members were limited to mediating between the community and BRTC and were thus not expected to undertake research-related roles including recruitment activities, ethical review of research (i.e. not an additional review board) or in the design of any project or trial. These CAB members included two representatives from each village including one male and one female. Their main duties were to inform the community on research activities, facilitating community members to participate in research related activities. They also helped in consenting processes as well as paying visit to the participants. These CAB members were project specific. Many challenges may be represented in the environment of low-income and low-resources countries, such as community expectation of financial assistance from CAB members. Nevertheless, these CAB members and village/community health workers may play health check roles. However, complex scientific concepts were communicated to audience who lack basic knowledge in science ^9^.

There is growing literature on community engagement across different research settings. Much of the documented evidence focuses on different approaches of engaging communities including different forms of CAB or Groups whose role is to advice researchers on the community they represent ^2^. However, there is very little evidence on functionality of the different structures that are involved in community engagement. Unfortunately, since the formulation of the CAB at IHI-Bagamoyo, we are not aware of works done to assess community structures in Ifakara as well as in Tanzania.

### Aim of the study

We assessed existing community structures and engagement processes used by researchers at IHI, to engage Community during health research, to explore perceptions and experiences of community stakeholders with regards to functioning of the community structure in facilitating engagement about health research and explore perceptions and experiences of IHI-researchers on the functioning of the current community structures in clinical research.

This descriptive qualitative study was designed to explore the nature of community structures/networks that could be engaged in health research at the Ifakara Research Centre, the strength and weakness of working with such community structures/weakness, and the impact of the structures on research conducted in the centre, including promoting research participation. In this paper, community structure/networks refer to community leaders/gatekeepers as earlier described. Engagement includes any form of involvement of these gatekeepers in research activities, including information sharing about research and consultations to get their inputs concerning on-going or/and planned research activities.

## Methods

### Study area

The study was situated in Bagamoyo district, Pwani region. The Bagamoyo study area has a total of three Divisions, eight Wards, and 24 village governments. The total population is 31,1740 people according to census of 2012 ^10^. The Ifakara Health Institute-Bagamoyo (IHI-Bagamoyo) branch is located on the grounds of the Bagamoyo District Hospital, on the coast of the Indian Ocean. The IHI-Bagamoyo employs several staff, including administrative support staff, clinicians and scientists ^9^. Diverse ethnic groups including the Zaramo, Kwere, Doe and Zigua inhabit the study area, and the majority are either subsistence farmers cultivating rice, maize and cassava or/and fisherman working on the Indian Ocean or the Ruvu River and its tributaries. Kiswahili, the national language, is spoken widely throughout the study area. This research focused at IHI-Bagamoyo center (found in Dunda ward) and on three randomly selected villages (Kongo, Buma and Matibwa). These villages are among 16 villages that are participating or participated in clinical research conducted by IHI-Bagamoyo within the last five years (2012–2017).

### Study design

This qualitative descriptive case study focused on one research site (IHI-Bagamoyo). Data were collected from February, 2019 to April, 2019. The study explored diverse stakeholders (communities and researchers) expressions/narration of their perceptions and experiences of working with the community structure/networks. The aim of the research approach was to generate textual information from the interviews with stakeholders, unpack range of community structures that are involved in the engagement process, determine the strength and weaknesses of these structures and provide recommendations on the engagement of IHI with the community hosting the research. It was expected that the data will provide both depth (detailed descriptions) and breadth (diverse views) around community engagement at IHI ^11^.

### Study respondents

A total of 55 respondents were invited to participate in this study and were divided into three groups. All respondents participated or were involved in IHI research. The first group included 36 community members (18 females and 18 males) from the three aforementioned villages; the second group included community structures, which comprised of three village executive officers (VEOs), three hamlet leaders (HLs) and three community health workers (CHWs); and the third group included researchers who constituted of three Principal investigators (PIs), two study coordinators (SCs), three project managers (PMs), and two field workers (FWs).

### Sample size selection

Purposive sampling was used in selecting the respondents. Purposive sampling was used to allow diversity and depth of views on community structures and thus included the different groups that are described above, to provide perspectives from community members participating in studies, the researchers and the community structures themselves. Village leaders of the selected villages were informed about the purpose of the study before the commencement of the data collection. The village leaders contacted the CHWs working in the selected villages, and the investigator invited them to participate in the study. The participants who were involved in IHI studies were identified by CHWs who were currently living in their village and were invited to participate in the study. Researchers who have been participating in IHI research for the past five years were identified and invited to participate in research by the PI of this research project. In the selection of respondents in each category, we considered those individuals that participated or are participating in IHI research between 2012 to 2017.

### Data collection

Two qualitative data collection methods were used. Focus group discussion (FGD) was chosen as it is useful in helping researchers learn about the social norms and perspectives that exist in the community and its subgroups ^12^. Furthermore, it can unpack community experiences (among those who participated in research) regarding the community structure that facilitated engagement in clinical research at IHI-Bagamoyo. In-depth interviews (IDIs) were used with the aim of exploring perceptions and personal experiences of the participants about the community structure in facilitating engagement in clinical research conducted by IHI in Bagamoyo.

Prior to data collection, two experienced research assistants were recruited and trained on the interview guides for IDIs and FGDs ^13^. The training was done in Kiswahili since data collection and guides were all developed in Kiswahili. The tools were first piloted with 10 respondents from the IHI and community members from Chasimba village. Piloting the tools aimed to test the appropriateness of the data collection tools, provide the researcher with some early suggestions on the feasibility of the research, and facilitated the researcher to obtain experience on data collection ^14^. Importantly, it assisted the researcher to learn critical interviewing skills and how to maintain a flow of conversation ^14^. The pilot phase included four IDIs and one FGD. The data from the pilot phase are not included in the final analysis. After piloting the tools, it was observed that some of the questions in the tools had to be adjusted for clarity; for example, in the interview guide for the FGD there were two questions addressing the same information, so one question was dropped.

Data were collected in Kiswahili language. Data collection process was flexible enough to allow participants to set the appointment dates. Six FGDs were held at local government office as well as at village dispensary. Furthermore, 49 IDIs with the community structures (VEOs, CHWs and HLs) and researchers (PIs, PMs, SCs and FWs) were held at their respective offices. The IDIs and FGDs were audio-recorded and approximate time for each interview was 45 minutes to one hour. The FGDs and IDIs were done by two research assistants, one research assistant was doing the interview and the second was taking some notes during data collection which were later converted into expanded notes ^15^.

### Data management and analysis

Data were transcribed verbatim independently by two researchers and translated from local language (Kiswahili) to English. Quality of the data were checked by the PI through listening of the tape recorder and reading of the expanded notes and debriefing report. The transcribed data from voice recording were read and re-read to gain initial impression of the data, and an in-depth understanding of participants’ description. We used both inductive approach (ideas emanating from the data itself) and deductive approach (theoretical understanding, literature review and researcher`s experience) for data analysis. Open coding framework was developed by reading the transcript and these codes were grouped in analytical themes. A framework matrix was developed based on these themes, the themes and matrix were reviewed by another researcher who had a background in social science. Findings were analyzed by comparing response across different groups in relation to their experiences and verbatim quotes were used to illustrate key themes ^16^.

### Ethics approval and consent to participate

Ethical clearance was obtained from the Institution Review Board of Ifakara Health Institute (approval number IHI/IRB/NO: 06-2019). An information sheet was drawn in Kiswahili explaining why the study is being carried, by whom and what it involved. Respondents were asked if they have any questions and whether they agree to take part in the study. Informed consent was obtained from each of the participants involved in this study, for the IDIs and FGDs. Responsible district authorities in which the study was to be taking place were informed beforehand to ensure support and security. Written informed consent was obtained from all study participants. Confidentiality of the patient information was guaranteed at all times, and data anonymity was considered during data collection procedures.

## Results

### Socio-demographic characteristics of respondents

Demographic characteristics of respondents are displayed in the Table 1. Overall, 55 respondents were interviewed, of which 45 were community members (including community networks/structures) and 10 were researchers. Of the 55 respondents, 45% were female while 55% were male. Most of the respondents reported having primary education. Furthermore, 11 of respondents including community leaders and researchers reported having a university degree. Age of most of the respondents was between 37 to 64 years; while a minority were aged 25 to 30 years.

**Table 1.** Demographic characteristics of participants.

Characteristic	Community members (n=36)	Community structures (n=9)	Researchers (n=10)	Total (n=55)
**Gender**				
Male	20	6	7	33
Female	16	3	3	22
**Age (years)**				
≤34	4	2	0	6
35 – 44	17	0	8	25
45 – 54	11	6	1	18
55 – 64	4	1	1	6
≥65	0	0	0	0
**Occupation**				
Farmers	6	0	0	6
Small scale businesses	28	0	0	28
Not employed	2	0	0	2
Community health workers	0	3	0	3
Village Executive Officers	0	3	0	3
Hamlet Leaders	0	3	0	3
Principal Investigators	0	0	3	3
Project Managers	0	0	3	3
Field Workers	0	0	2	2
Study coordinators	0	0	2	2
**Education level**				
Did not attend school	6			6
Primary school	30	7	1	38
University 1 ^st^ degree	0	2	2	4
University 2 ^nd^ degree	0	0	5	5
University 3 ^rd^ degree	0	0	2	2

### Existing community structures/networks engaged by IHI researchers

Findings from the interviews indicate that different community networks/structures have been involved by researchers in Bagamoyo, during the implementation of research related activities. Researchers described community networks/structures as consisting of village government, which comprised of ward executive officers, village chairmen, village executive officers (VEOs), hamlet leaders (HLs) and community health workers (CHWs). In addition, it was reported that these networks were more aware of their community and the context where the specific research was happening. They further have extensive knowledge of their community dynamics including community needs and what would be community response towards a particular research. This insider knowledge is important because it can inform researchers about the important social-cultural issues that they need to take into account during conducting their research. It also became apparent in the interviews that some of these structures are trusted in the community (e.g. VEOs, HLs and CHWs); thus, it is easier for researchers to enter in particular village/community and access respondents for specific projects through that networks/structures.

          “ *In Bagamoyo more specifically at IHI, there are different structures or levels which are involved to reach the community, of which researchers must pass through. These are village government, community health workers. There is also a board which I have heard however, I have never worked with it. It is known as Community Advisory Board. This board was used previously but since I started working in Bagamoyo almost a year now, I have never met any member of the board.*” ( ***PI (1), male, IDI)***

However, at village government level there are different social service boards (known as CABs), which are responsible in advising the villagers on different aspects related to health, environment and education. Researchers reported that based on the type of research, the village government leaders can link researchers to a particular board based on their need. This may help in gathering people for public meetings to be informed of the expected study project. For example, since IHI’s main role is conducting health research, at a village government level, IHI researchers can be linked with community advisory board involved in health-related activities, of whom community health workers are part of it. A PM reported this as follows:

*“In the village government, there are different boards such as social service board, boards related to health, water and education. So if there is any research project, at the village government they connect us with specific board, that board will talk with chairmen and gather a village meeting*” ( ***PM (1), male, IDI***)

It has also been reported that some of the community networks/structures that are often engaged by IHI researchers are project specific. Some of the projects recruit respondents from healthcare facilities. Patients seeking health care in these facilities may be sensitized about IHI studies by nurses, doctors or people from the research team. Additionally, CHWs are involved at the village dispensary level in engaging with community members who happen to visit the dispensary. Moreover, there are routine health care campaigns in the community to create awareness of the particular diseases, such as tuberculosis day (TB day), malaria day and HIV day; sometimes researchers use these platforms/campaigns to facilitate engagement around specific research project:

          “ *There are different mechanisms used in the hospital to sensitize patients who come at the hospital as normal routine for treatment. But also, community health workers are involved to engage the community at village dispensaries or households. There are campaigns such as for TB, Malaria and HIV day. These platforms are used to engage the community. In short, the community can be engaged when patients visit hospitals for normal routine and during the campaigns for diseases like TB.” ( **SC (2), male, IDI)***

          “ *Most of the time apart from Community Advisory Boards, we have been using government system, as for me, I do research related to Tuberculosis, so I use District Coordinators for Tuberculosis, nurses of the specific area to patients with Tuberculosi*s” ( ***PI (2), male, IDI)***

### Approaches used by community structures/networks during engagement activities

Several approaches were reported to be used by the community networks/structures when engaging the community about IHI research projects. The approach used appeared to depend on several factors including; the type of research that communities are being engaged about; the study criteria in selection of participants; and the nature of information that will be provided. The approach used included public meetings, which were open for everyone to attend for general sharing of information about the general activities of IHI, and introduction of a team or research study in the area. Household visits by HLs and CHWs were used for deeper conversation at the household level about a particular research, where potential participants could be recruited from the home. Sensitizing patients who visit certain Health facility be it Dispensary or Hospital, was done at the health facility level.

*“… there is CAB or community service board; they gather a meeting and talk to the community, those CAB members used to go to certain family, communicate general information to the villagers or Hamlet leaders” ( **PM(2), Male, IDI**)*

          “ *At the Hospital, patients go to seek service. He/she will meet with health service provider and if diagnosed with Tuberculosis, she/he will be informed of a research project which is going on, when agreed Health provider will inform us, we go and take them or if it is nearby they bring him/her*”( ***PI (2), male, IDI***)

### Advantages of involving community networks/structures in research

Researchers reported that the advantages of the community structures were linked to the support that researchers received while conducting research in a particular community. The support helped in increasing the community trust towards the research team, subsequently contributing to the researchers meeting their research goals. It was further suggested that the community structures were most helpful during recruitment phase of the studies, as they helped in solving conflicts among the community regarding research projects: 

          “ *Community structures have been doing very great job specifically during Malaria vaccine trial (RTS,S), because it was a new vaccine and it had a lot of challenges. But through structures we did not experience rejection in the Community, problems and rumours in the Community were solved. Village government cooperated with us effectively*” ( ***PM (2), male, IDI)***

          “ *, …….. simplicity in doing the work, acceptance of the research team and research in general, and getting participant easily are other advantages of the community structures*” ( ***PI (2), male, IDI***)

### Views/opinions on the community engagement activities

There were different opinions that were reported regarding community engagement processes, and activities that have been used to engage the communities about IHI research projects. Community members reported the involvement of the community structures/networks during engagement activities is very important, simply because they have to be informed of what researchers want to do. They suggested that processes of informing the community can be improved to a point of using media such as radio, television, posting announcement so that people can be aware of what is going on around the community.

          “ *Other information regarding research should be given through media, posting announcement. But going through community structures is the proper way because they have to know any project that comes in the village” ( **CR (12), male, FGD**)*

We found that researchers should also find time to visit areas where community structure have been sharing information on research activities. These will therefore emphasis important elements of the information, and to clarify some of the areas that are difficult for community members to understand.

*“…...there should be close communication between CAB members, village government and researchers. Researchers should frequently visit the community to emphasis on what have been said by the community structures. there may be discrepancies on information shared in more than one village” ( **PM (1), male, IDI)***

Researchers also reported that it is important to develop a list of frequently asked questions and find a way of simplifying scientific words in a language that can be understood by people of all levels. This will help in elaborating the different aspects related to research activities.

*“……Develop list frequently asked questions and create answers and be given to those who are very close to the community. For those which have no answers, they should make reference from researchers” (PM **(1), male, IDI)***

Researchers also recommended strengthening the department of community engagement at all branches of IHI, by establishing reliable and strong community advisory boards. Resources should be allocated in this department for it to work effectively. In addition, there should be relationship between researchers and the communities whereby researchers can contribute in community activities like building of the dispensaries, schools and IHI doctors can also work with district hospitals and help in providing treatment services. This is one way of maintaining regular contact with the communities.

          “ *Sustainable way, I think is to build strong CAB which will strengthen department of community engagement in all IHI branches.. there are still some gaps, I think because resources have not been allocated, these will help to build relationship and establish engagement with the community” ( **PI (1), male, IDI**)*

           “ *It is good to have regular contact with them, if there is anything their doing in the community can collaborate with them, like building of dispensary, schools it give good relationship, for example IHI doctors can collaborate and involve in treatment activities at the District Hospital, because people are the same and even if we go they recognize us, it help us to be known as Institute to the community.*”( ***PI (3), Female, IDI***)

## Discussion

From the findings described above, several areas emerge that are related to the role of community structures in facilitating community engagement at IHI, the role that community structures play in engagement and how they are perceived by the researchers and the community, and the recommendations to IHI with regards to community engagement during research activities. We describe these areas below.

### Role of community structures in facilitating community engagement at IHI

From the findings, it appears that IHI researchers chose different types of community structures to engage with community about research projects. The choice depends on the types of the research, as well as the kind of participants that are needed for specific projects. Community engagement at IHI have involved a range of community networks or structures. These include ward executive officers, VEOS, HLS, CHWs, community advisory boards, and nurses and doctors at public health facilities. This is important so as to maintain the interest of the community in the IHI work, even when there are few ongoing projects, as the research projects depend on the participation of the community members. It is also notable that different community structures are used in the community. The use of several structures seems to have worked well, since it appears that the tensions reported from the use of one structure in a Kenyan study ^2^ did not emerge in this context. In addition, it seems that identifying the community structures through the village government office is one way of recognizing the central role of the local community leadership, and may have demonstrated respect and cultural humility towards the community ^17^.

The results of this study also suggest that the respondents felt that relations between researchers and community structures are skewed towards the researchers’ interest and needs. Thus, there are closer relations during study sensitization processes, whereby community structures facilitate members of the particular villages to engage in health research. However, once these studies come to an end the relations also seem to wither with no further engagement or communication between researchers and community structures. Given that IHI is likely to be in the community for a long period of time, it is important to re-think how best engagement with the community can be sustained. Individual research projects may not be able to sustain continued engagement with a particular community or community structure beyond the study period due to budget/funding limitations. However, the Institute can make a case to funders for a centralized coordinated engagement that can be funded at programme level, with research budgets supplementing for study-specific types of engagement. This would be one approach to sustain engagement, given the value such engagement seems to have in terms of building appropriate relations with the community participating in research, a relation that can facilitate the research to thrive in this setting.

### Improvement of community engagement activities

Different advantages associated with engagement processes were reported. These were assurance of the safety of the community members about certain research projects, as this helped members of the community to trust researchers and the activities associated with research. Being community gatekeepers, permission of a study by community structures was said to be important in gaining entry to the community and in reassuring communities of the goodwill of the researchers. However, the improvement of the engagement processes were suggested by the community members, which are additional to the previously used method of conducting general public meeting of which most of the community members hardly attended; and the few that attended those meetings, found it difficult to grasp all the information regarding a certain research. Additional complementary engagement activities suggested include visits at household of the community members to meet head of the house and other family members, use of radios, television, posting announcement at places where people tend to gather for different purposes like in the market places, football play grounds, bars and restaurants. These additional methods are likely to emphasize what have been communicated by the other method and will help community members to understand more when different method are used to inform them of the certain research project to be initiated in that area.

The findings also described the importance of simplifying difficult scientific words in a way that makes it easy for community to understand. This goes along with the compilation of questions and answers for the frequently asked questions to ease the communication with the community structures during the engagement activities. This suggestion emanated from the observation that community structures often failed to communicate same message to several villages without distorting the meaning. This can contribute to miscommunication of the particular research purpose. This is because often health research involves consideration of information, unfamiliar process and terminologies that require repeated sessions of information-giving ^6^. In addition, and to support good engagement processes, it was suggested that adequate funds in a budget should be allocated to strengthen the community engagement department. The ethical importance of community engagement is underpinned by its value in identifying socially and culturally appropriate consent processes and ethical conduct of different types of studies. Therefore having a well-established community engagement system will help researchers know what the needs of the community are, and how best these can be addressed, and create relations of mutual trust and respect between researchers, community and community structure.

### Limitation of the study

Our research findings are not without limitations. First, we were not able to capture data in urban community due to time and budget constraints. Furthermore, the research was not able to capture data of the community members who have never participated in IHI research due to time constraints. Therefore, perceptions and experiences from these two groups on the systematic functioning of the community network, were not captured so as to have comparable data from rural and urban areas. The data would have helped to have a wide understanding of the perceptions and experiences towards community networks/structures that have been facilitating engagement in health research

## Conclusion

This informative research was conducted to understand the systematic functioning of existing community networks/structures in facilitating engagement in health research. Findings reported different aspects related to the role of community structures in facilitating engagement and the type of engagement. The community structures involved in the engagement seemed to have been influenced by the type of research project and kind of participants needed. Proposed ways of strengthening engagement with the community and researchers included use of various media of engagement such as radio, television, posters and regular meetings with the community during the course of a study. Subsequently, this will enhance understanding of the research information by the community members and community structures, as well as help raise some of the issues that could be considered when developing a research protocol. Frequent interactions will also enhance information sharing with the targeted population. This can be done by developing a list of frequently asked questions and answers so as to facilitate the understanding of the community stakeholders. A system of giving feedback to the community regarding the research work done by the IHI should be considered. Finally, it is important that there is central coordination of the community engagement work at IHI. This requires development of a community engagement unit that would work across projects to support them with the engagement in the community. The unit will maintain continuous engagement with the community and conduct research to understand the relationship between communities we work in and researchers. Funding of this unit could be done through contributions from the core budget, individual’s projects or competitive grant application.

## List of abbreviations and symbols

BRTC     -     Bagamoyo research and training center

CAB      -     Community advisory board

CDC      -     Center for disease control

CERC      -   Community engagement research core

CES      -     Community engagement studio

FGD      -     Focus group discussion 

HIV     -     Human Immunodeficiency syndrome

IDI      -     In-depth interview

IHI      -     Ifakara Health Institute

IRB      -     Institution review board

CHW      -     Community health workers

KEMRI      -     Kenya medical research Institute

DED      -     District executive director

NIMR      -     National Institutes of Medical Research

PI     -     Principal Investigator

PM     -     Project Manager

SC     -     Study Coordinator

FW     -     Field worker

HL      -     Hamlet leader

CR      -     Community respondent

VEO     -     Village executive officer

## Data availability

### Underlying data

According to the Institutional Review Board (IRB) of the Ifakara Health Institute, we are not allowed to make the transcript data from the focus group discussions or the in-depth interviews publicly available due to fact that policy for data circulation has to be signed. Quotes from the transcripts are provided in the text as intermediary data. Interested researchers should contact the corresponding author or irb-submission@ihi.or.tz. Data access will be granted under the following conditions: (i) signing data sharing agreement; (ii) waiver of the informed consents by IRB if the justifications are considered to be ethically and scientifically sound, since the consent forms clearly stated that the participants’ data will not be shared outside the Institute. 

### Extended data

Figshare: Community-networks that facilitate engagement in health research: Ifakara Health Research Institute-Bagamoyo case study, https://doi.org/10.6084/m9.figshare.14113229.v1 ^13^.

This project contains the following extended data:

- Community networks_Community stakeholders interview guide,
- Community networks_Researchers interview guide
- Community_networks Group discussion guide.up

Data are available under the terms of the Creative Commons Zero “No rights reserved” data waiver (CC0 1.0 Public domain dedication).
