# Community-structures that facilitate engagement in health research: Ifakara Health Research Institute-Bagamoyo case study

**DOI:** 10.12688/aasopenres.13187.2

**Published:** 2022-03-23

**Authors:** Leah Bategereza, Ally Olotu, Dorcas Kamuya

**Affiliations:** 1 Ifakara Health Institute, Bagamoyo, Tanzania; 2 School of Life Science and Bio-engineering, The Nelson Mandela African Institution of Science and Technology, Arusha, Tanzania; 3 Kenya Medical Research Institute (KEMRI), Kilifi, Kenya

**Keywords:** Community networks/structures, community engagement, health research

## Abstract

Background: Involvement of communities in health research has been at the forefront of ethical conduct of research worldwide. Despite the fact that many scholars have put forward different ways of engaging communities in health research, debates on the forms and goals of engagement, levels of engagement, who should be engaged in the community and how, still persist. At the Ifakara Health Research Institute (IHI) in Bagamoyo, Tanzania, different approaches to engaging with the community in health research have been used over the last decade. Of the approaches that have been used, are the use of community structures including traditional and administrative leaders, which have been periodically engaged, but with little sharing beyond study-level. Therefore, the present research was aimed to describe the nature of community structures that could be engaged in health research at the Ifakara Research Centre, the strength and weakness of working with such community structures, and the impact of the structures on research conducted in the center, including promoting research participation.

Methods: A qualitative study based on social science methodological design and a thematic approach for data analysis was employed. Data collection was undertaken in between February 2019 and December 2019. In this study, a total of 25 interviews with 55 respondents in which 19 were In-Depth Interviews (IDIs), and 6 were Focus Group Discussions (FGDs) were carried out. The In-Depth Interviews (IDIs) involved Village Executive Officers (VEOs), Hamlet Leaders (HLs), Community Health Workers (CHWs), Principal Investigators (PIs), study coordinators (SCs), research project managers (PMs), and field workers (FWs), while 6 Focus Group Discussions (FGDs) involved community respondents who previously participated in IHI research. The FGDs were conducted in 3 villages; two FGDs in each village, one each for men and women. The interviews were audiotaped, transcribed, and analyzed using framework analysis. Comparative thematic analysis was undertaken as more data was added, creating new themes, until there was no new emerging themes, a point of data saturation. The themes were charted across respondent groups to map patterns of the themes across respondents groups.

Results: Data shows different community structures including the village executive officers, community health workers, hamlet leaders, and community advisory board were involved in engagement activities. Approaches used for engagement as per the findings under this study include community-level public meetings, information giving at household level and at the health facilities, the use of outpatient attendance at hospital/dispensary, the use of Health District Coordinators, the use of some members of village leaders/representatives families through their both informed assent and consent as project or research participants, and the use of routine health care campaigns in the community to create awareness of the particular diseases such as Tuberculosis day (TB day), Malaria day and HIV day. The weaknesses that were reported include inconsistence research feedback, insufficient engagement with participants about specific research projects they are recruited into and false promises by researchers to community stakeholders. Community stakeholders suggested additional ways to engage with the community, these include radio, advertisement, posters, brochures and regular meetings.

## Introduction

### Community engagement and its roles in health research

The term community can be defined as a group of people that interact and support each other, may share common goals and interests, could be bounded by shared experiences or characteristics, a sense of belonging, and may share physical proximity ^1– 5^. Community engagement (CE) refers to the process of working collaboratively with and through groups of people affiliated by geographic proximity, special interest, or similar situations to address issues affecting the well-being of those people ^6^. Community engagement is also often be described as a spectrum, ranging from information giving, consultations, involvement in research activities, and to higher levels of partnerships where community members are involved in research decision-making, for example as co-investigators in research activities ^6– 8^. Additionally, community engagement in health research is described as a collaborative relationship between researchers and communities hosting the research in which there is mutual understanding and agreement on the goals of the research, and that community have a sense of ownership towards the research and achieving its goals ^9^.

Researchers have described several ethical goals for engaging communities in health research. These include: To protect the community through considering a fair balance of benefits and risks for different types of research, strengthening fair selection of research participants, and minimizing risks and burdens in research participation ^10^. In addition, community engagement can contribute to addressing some of the ethical concerns for conducting research particularly in developing countries. These include: (a) Minimizing potential for exploitation (b) Generating research that has fair benefits (c) Creating awareness and respect of cultural differences between researchers and communities (d) Promoting respect of recruited participants (e) Minimizing community disruption, and (f) Minimizing of the health and wealth disparities, inequalities and stigma (evidence of changed norms and behavior around disease related) in the community through community engagement ^10^.

Community engagement is purported to be an important element of ethical research particularly in Sub-Saharan Africa as it demonstrates respect, as well as can strengthen: Informed consent processes, the science of the research and can inform on community values that researchers ought to consider ^10^. Moreover, community engagement is becoming a common component promoted by a number of research institutions and funding bodies ^11^. On the other hand, community engagement has been allowing marginalized voices and experiences to be represented in the production of scientific knowledge while ensuring the relevance of research as well as impactful ^11^. Subsequently, community engagement should be focusing in creating a meaningful partnership between researchers and those that inhabit in the social or physical space where research is being conducted. Because of its impact on the conduct of health research, community engagement is now being regarded as one of the important aspects to be considered in National guidelines for clinical research ^10^. Besides, some of the key health research funders recognize the important contribution of community engagement in health research; for example, the Wellcome Trust expounds on the role of engagement in advancing health research, set dedicated funding stream for engagement, and galvanized a call to other funders to join in spearheading engagement ^12^. They make a concerted call to other research funders to pay attention to and invest in engagement, noting that funders focus mostly on investing millions of dollars on product development, clinical training, designing and building of facilities, but have often ignored the important part of community engagement.

Despite all the aforementioned benefits of community engagement in health research, still there are more challenges that need be taken into account in order to full capture the essence and benefits of community engagement as far as the conduct of health research is concerned. This is more clearly substantiated in one major study on community engagement in facilitating engagement in biomedical HIV preventions ^13^. According to the findings under the said study three main challenges were identified. The first one is trial literacy; that includes lexicon challenges and misconceptions which imperil sound communication. Therefore, as a means to bypass this challenge it is necessary to educate the community stakeholders about the key issues within the context of proposed research. The second one is mistrust due to historical exploitation and participatory processes. With this one, it was reported that community perceptions and conceptualizations of clinical trial research in particular is predominantly affected by the historical experiences with colonialism, marginalization, and exploitation. Therefore, experiential knowledge through face-to-face communication, including testimonials from former trial participants is necessary to regain trust with researchers. Additionally, evidence of trials from abroad needs to be communicated to the community before local implementation. This is more important specifically to clear the common doubt that local and disadvantaged communities are more often used as guinea pig for clinical trials. Also, researchers and trialists need to clearly explain the chance of early trial termination in case of any unexpected outcomes. The last reported challenge by the said study is meaningful community stakeholder engagement whereby it is emphasized that early meaningful engagement with community stakeholders is necessary during trial planning process, protocol formulation and to determine community perception on the social value of the research, and should extend to include a life time of a trial. Early engagement was thought to minimize historical mistrust as community stakeholders would feel that they were part of the research activity, and thus identified with some partial ownership and control of the research.

While the ethical goals for community engagement are well recognized ^14^, the forms and levels of community engagement depend on a number of factors such include the issue/topic of engagement and the context dependent. Forms of community engagement could include information-giving to high levels of involvement, participation, consultation and collaboration ^15^. Most community engagements are undertaken on studies that have been approved by research ethical committee ^16^. Such engagements have largely been framed as meeting instrumental goals of ensuring that the research is acceptable and that recruitment targets are met and sustained. Furthermore, there is greater understanding that community engagement also has intrinsic value that is, it is important to engage communities because it is the right thing to do.

In clinical research, the involvement of the community has largely been framed as instrumental, e.g. in order to meet recruitment and retention targets, to strengthen consent processes, address issues as they emerge etc. As collaborative international research increases, involvement of diverse range of community representatives and participants also is important for broad buy-in of the studies and to ensure that stakeholder interests feed into the conduct of the studies wherever possible ^17^.

### Potential involvement of community structures in health research

Community structures can be explained as relevant groups in a community of which the researchers consult before meeting an individual for informed consent, and seeking for permission before approaching members of the community, such groups are community leaders, administrative leaders, religious leaders, ethical committee, community representatives and community advisory boards. These structures help the research team in getting insight on the cultural values that may have an implication on the research. They can also enlighten the priority health needs of the community, which might contribute to a better understanding of locally-relevant research ^2^. In certain circumstances, they can make decisions on behalf of the communities they lead, and those decisions can be binding to their members ^18^. Community representatives can work with research staff, and be provided with opportunities to be involved in overall activities regarding the research such as development of the research protocol, obtaining consent, collecting and reviewing data, access to data and sampling ^19^.

In Africa for instance, several studies have documented evidence of involving community structures in health research. In South Africa, for example, a study by Musesengwa *et al*. employed both Community Advisory Boards together with direct advice from community leaders and community liaison officers to facilitate the community entry and obtaining letters of permission ^20^. Furthermore, in this study it was concluded that engaging community in health research should be consistent with community values and attitudes, and consider community resources and capacity. However, the study also concluded that, although engaging community in health research creates a conducive research environment, the engaging process is always faced with challenges such as community research literacy levels, time and resources. Another study by Tindana *et al*. in Ghana employed traditional pathways of engagement and decision to get entry into community ^21^. According to this study, preexisting features of the community such as clear channels of authority, progressive layers of accountability, processes for assembling the community, and processes for deliberating new ideas and proposals greatly facilitate community engagement in health research. Therefore, from this study researchers are supposed to seek permission from chiefs and leaders of the community before contacting the community members. Furthermore, researchers should rely on traditional chiefs in calling for a large-scale gathering; allows them to fulfill their traditional stewardship roles as leaders and protectors of the community. A study conducted in Kilifi, Kenya to describe the complex realities on community engagement for a paediatric randomized controlled malaria vaccine trial showed that field staff and community leaders (chiefs, village elders, community health workers (CHW), health facility staff and health committees) were generally considered by the research team as the channel for community members to voice any concerns. Furthermore, in the same study it was reported that involving community leaders in that study helped clear pre-existing concerns and misconceptions about Kenya Medical Research Institute (KEMRI’s) work; raised awareness; built trust; and increased the visibility of KEMRI staff initially seen as ‘outsiders ^22^. In western Kenya, a large well established Kenya Medical Research Institute in collaboration with the US Centers for Disease Control (KEMRI/CDC), involved community members; referred to as village reporters – in their research activities ^23^. Their involvement was later formalized through the development of standard operating procedures that clearly stated their roles and renamed them as village reporters. Being embedded within their community seemed to solidify their ability to create good working relations and exposure gained from KEMRI/CDC in terms of training and meetings strengthened their understanding about research ^23^. Reported challenges with this system include different reimbursement rates across different studies to the village reporters which created tensions between the village reporters and across different study Principle Investigators ^23^.

Another study on using traditional community assemblies to investigate community perspectives on informed consent and research participation in western Kenya, opted to use Mabaraza which are officially organized by a Chief, or sub-Chief, and other village leaders so as to get entry to community members for their consent and participation to the research activities ^24^. In this study it was concluded that Mabaraza in East Africa should be used as a forum to further engage communities in community consent and other aspects of research ^24^. In a study that investigated community perceptions of mass screening and treatment for malaria in Siaya County, western Kenya, village leaders were the key community structures, which were fully involved from preliminary sensitization process to recruitment, and finally data collection processes ^25^. Based on the findings of this study, it is concluded that a more long-term approach to community engagement is necessary for dispelling fears, clarifying misconceptions pertaining the research goals ^25^. Another study on Recruitment of Yoruba families for a multisite keloid study utilized a modified community-based participatory research approach to engage keloid patients and the community at large as follows: First, this study made use of former keloid patients as patient adviser with the main role of sensitizing and educating the patients with keloid to take part in recruitment process. However, this study involved a number of community structures such that; king, chiefs and ward leaders as village leaders; Medical and nursing directors of local health centers and private hospitals as health directors; and pastors and imams as religious leaders ^26^. Furthermore, this study used approaches such as; advertisement through handbills and posters in both Yoruba and English language in the communities specifically in market places, private and public health clinics, schools, churches, pharmacies and clinical laboratories; radio and TV presentations in English and Yoruba language describing the purpose of the study, eligibility criteria and contact information; and the use of affected/enrolled participants with keloids to spread word about upcoming recruitment visits in their community. Despite an extensive use of community structures to engage community members to participate to the research, the study reported some challenges it faced during recruitment process. These include infrastructural shortcomings, cultural and religious beliefs, which can lead to significant delays, and may negatively affect study time lines and expectations of funding agencies ^26^. In a study conducted in Mwanza-Tanzania on Microbicides Development Programme (MDP301 trial), a range of community structures were employed for the sake of getting community members to positively respond to the project. These included: Community liaison Officer (CLO), a Community Advisory Committee (CAC) and object-specific Stake-holders Advisory Group (SAG). Each of these had a role to play in the trial as follows: The CLO facilitated open dialogue and partnership working practices between researchers, study participants and community representatives, both CAC and CLO facilitated the formation of Cluster, ward and city-level representatives, while SAG acted as advisor for the CAC ^27^. Although community engagement is recognized as intrinsically respecting and protecting communities, in some instances depending on the nature of a research it has to go beyond community level. For instance, a study about rules of engagement on perspectives on stakeholder engagement for genomic bio banking research in South Africa, different layers of engagement were identified such that: High level (policy makers, institutions and funders), Peer level (scientists, medical doctors, bio bankers, nurses and field workers) and Community level (community members, patients, research participants, support groups, field workers and CABs) ^28^. Based on this study, the engagement of each stakeholder layer is important to ensure the success of genomic bio banking research. In the same study, it is further concluded that despite the fact that community engagement is time-consuming and often requires an interdisciplinary research team approach, careful planning of the engagement strategy, including an understanding of the differing layers of stakeholder engagement, and the specific educational needs at each layer, can help in the development of a relationship based on trust between the research team and various stakeholder groups ^28^.

### The ifakara health institute-Bagamoyo

Bagamoyo branch of the Ifakara Health Institute (IHI-Bagamoyo) was initiated in 2005 to conduct clinical research in Bagamoyo District, Tanzania. Subsequently, many (including over 23 clinical trials) ground-breaking clinical research studies have been conducted by the branch, including studies investigating Coarterm Pediatrics formulation, Phase III trial assessing the efficacy of a most advanced malaria vaccine candidate, RTS, S, and TB drug trials. Of the 23 trials conducted so far, nine were malaria vaccine trials, six were malaria drugs trials, two were TB vaccine trials, four were TB drug trials, one was a micronutrient (Iron) supplementation trial, and three were diagnostics trials. All these clinical trials required the participation of the community members as participants ^29^.

### The Community Advisory Board (CAB) at ifakara health institute

In 2006, a Community Advisory Board (CAB) was established to work alongside with Bagamoyo Research and Training Centre (BRTC) on research-related activities involving the community. Its role was to mediate the fair balance regarding research benefits and risks between the community and the researchers, and inform researchers about community concerns with regards to their research/projects. The CAB was also involved in dissemination of information about planned research, and facilitation of interactions between BRTC and the community. However, CAB members were not expected to undertake research-related roles such as recruitment activities and review of research. CAB members were drawn from the community, with each village selecting 2 members ^29^. Through the CAB, IHI was able to address concerns in the community with regards to study activities, for instance, concerns such that too much blood was collected from participating children. As has been reported in the previous studies above, some of the challenges identified with working with CAB at IHI members included high expectations of immediate direct benefits for themselves and their families, community expectation of financial assistance from CAB members, among others ^29^. In addition, the CAB at IHI was set-up as a project mechanism with no proper central coordination, and its sustainability is dependent on continued funding of the projects. Once these projects end, it is not clear if the CAB will still be continuing functioning as there is very little documentation of the process at the time of setting up this project. Therefore, this study was designed to explore the nature of existing community structures (including CAB members) that could be engaged in health research at the Ifakara Health Institute, the approaches used by the IHI research center, research teams/ PIs/researchers to effectively engage the community into health research/project activities, the strength and weakness of working with such community structures, and the nature of existed relations among the IHI research center, research teams/ PIs/researchers and the community structures. We defined engagement to include any form of involvement of these gatekeepers in research activities, including information sharing and consultations to get their inputs concerning on-going or/and planned research activities.

## Methods

### Study area

This study was conducted in Bagamoyo district, Pwani region, which has a population of 31,1740 according to census of 2012 ^30^. The Bagamoyo district consists of a total of three Divisions, eight Wards, and 24 Village governments. The Ifakara Health Institute branch (IHI-Bagamoyo) is located in Bagamoyo District Hospital, at the coast of the Indian Ocean. The IHI-Bagamoyo employs several staff, including administrative support staff, clinicians and scientists ^29^. Diverse ethnic groups including the Zaramo, Kwere, Doe and Zigua inhabit Bagamoyo district, and the majority are either subsistence farmers cultivating rice, maize and cassava or/and fisherman working on the Indian Ocean or the Ruvu River and its tributaries. Swahili, the national language, is spoken widely throughout the study area. This research focused at IHI-Bagamoyo center (found in Dunda ward) and on three randomly selected villages (Kongo, Buma and Matibwa). These villages are among 16 villages that are participating or participated in clinical research conducted by IHI-Bagamoyo within the last five years (2012–2017).

### Study design

A qualitative study case design was deemed appropriate for this research as it generates rich textual information from interviews with the various stakeholders and thus, draws on individual narration of the lived experiences and perception of the phenomenon under study ^31^. In this study, such an approach allowed us to unpack: The range of community structures that are involved in engagement at IHI, the strengths and weaknesses of these structures and recommendations for engagement between IHI with the community hosting its researches. Data were collected from February, 2019 to April, 2019.

### Selection of respondents

Purposive sampling was used in selecting the respondents. This study employed this sampling design in order to allow for diversity and depth of views on community structures. Before selection of respondents began, the study population was categorized into three groups namely: Ordinary community members, Community leaders/ representatives and Researchers/ Researcher coordinators/ Researcher managers/ Field workers/ Principle investigators (PIs) at IHI-Bagamoyo center. The selection criteria for respondents was being participated in IHI research activities in the last five years (2012–2017). The respondents were selected from the preformed three groups as follows: 36 community members (18 females and 18 males) from three villages namely: Kongo, Buma and Matibwa; 9 members of community leaders/representatives such that three members from each of the followings: village executive officers (VEOs), Hamlet leaders (HLs) and Community Health workers (CHWs); and 10 research staff consisting of three Principal investigators (PIs), two study coordinators (SCs), three research project managers (PMs), and two field workers (FWs). In each of the selected village the study team first, introduced the study to the village leaders. In consultations with the village leaders and the community health worker representatives, potential community respondents were identified and invited to participate into the study. The research staff were identified in consultation with PIs. Informed consent were sought from all the potential participants. Data was only collected from those who gave consent.

### Tools development and data collection

Two qualitative data collection tools namely Focus Group Discussion (FGD) and In-Depth Interviews (IDIs) were used. Focus Group Discussion (FGD) was chosen as it is useful in helping researchers to learn about the social norms and perspectives that exist in the community and its subgroups ^32^. Furthermore, it can unpack community experiences (among those who participated in research) regarding the community structures that facilitated engagement in clinical research at IHI-Bagamoyo. In-depth interviews (IDIs) were used with the aim of exploring perceptions and personal experiences of the participants about the community structures in facilitating engagement in clinical research conducted by IHI in Bagamoyo.

Prior to data collection, two experienced research assistants were recruited and trained on the interview guides for IDIs and FGDs. The training was conducted in Swahili because data collection and guides were all developed in Swahili. The tools were piloted in four IDIs and one FGD involving six respondents. Piloting the tools was aimed to check the clarity and appropriateness of the questions, and providing the researcher with some early suggestions on the feasibility of the research. Importantly, it assisted the research team to practice critical interviewing skills and how to maintain a flow of conversation ^33^. The tools were revised including excluding questions that were similar or were asking for the same issues, providing further clarifications of questions that were not clear. Since the main aim of the pilot was to check for clarity of the questions, the data collected was not included in the final analysis of the present study.

Data collection included six FGDs involving 36 community members and 9 IDIs with the community leaders/representatives (VEOs, CHWs and HLs) which were held at local government offices and village dispensary; and 10 IDIs with researchers (PIs, PMs, SCs and FWs) held at their respective offices. Two research assistant conducted the interviews, one moderated the interviews supported by the LB while the second one took notes. Each interview took about 45 minutes to one hour. All the interviews were audio-recorded and were later transcribed ^34^.

### Data management and analysis

Data were independently transcribed verbatim by two researchers and translated from local language (Swahili) to English. Quality of the data were checked by the PI through listening of the tape recorder and reading of the expanded notes and debriefing reports. The transcribed data from voice recording were read and re-read to gain initial impression of the data, and an in-depth understanding of participants’ description. We used both inductive approach (ideas emanating from the data itself) and deductive approach (theoretical understanding, literature review and researcher`s experience) in data analysis. Open coding framework was developed by reading the transcript, and these codes were grouped into analytical themes. A framework matrix was developed based on these themes, the themes and matrixes were reviewed by another researcher who had a background in social science. Findings were analyzed by comparing responses across different groups in relation to their experiences and verbatim quotes ^35^.

### Ethics approval and consent to participate

Ethical clearance was obtained from the Institution Review Board of Ifakara Health Institute (approval number IHI/IRB/NO: 06–2019). An information sheet was drawn in Swahili language explaining why the study is being carried, by whom and what it involved. Respondents were asked whether they had any question and agreed to take part in the study. A written informed consent was obtained from each participant before involvement into this study. Responsible district authorities under which the present study was to be undertaken were informed beforehand to ensure support and security. Confidentiality of the participant information was guaranteed at all times, and data anonymity was considered during data collection procedures.

## Findings

### Socio-demographic characteristics of respondents

Table 1: Shows the demographic characteristics of respondents in this study. Overall, 55 respondents were interviewed of which 65.4% (n=36) were community members, 16.4% (n=9) were community leaders/ representatives and 18.2% (n=10) were researchers. Based on gender, Males 60% (n=33) were more by 11% than females (40% (n=22)). Of all males (60% (n=33)) interviewed; 36.4% (n=20) were community members, 10.9 % (n=6) were community leaders/representatives, 12.7% (n=7) were researchers. While of all females (40% (n=22)) interviewed; 29% (n=16) were community members, 5.5% (n=3) were community leaders/ representatives and 5.5% (n=3) were researchers. Of all respondents; 10.9% (n=6) were less than 34 years, 45.5% (n=25) were in between 35 to 44 years, 32.7% (n=18) were in between 45 to 54 years and 10.9% (n=6) were in between 55 to 64 years. Majority of respondents aged in between 35 to 44 years. Based on occupation and education level majority of respondents were in small scale businesses class (Entrepreneurs 50.9% (n=28)) and possessed primary education (69.1% (n=38)) as the highest level of education.

**Table 1.** Table 1: Demographic characteristics of participants.

Characteristic	Community members (n=36)	Community leaders/Representatives (n=9)	Researchers (n=10)	Total (n=55)
	n/%	n/%	n/%	n/%
**Gender**				
Male	20 (36.4)	6 (10.9)	7 (12.7)	33 (60)
Female	16 (29)	3 (5.5)	3 (5.5)	22 (40)
**Age (years)**				
≤34	4 (7.3)	2 (3.6)	0 (0)	6 (10.9)
35 – 44	17 (30.9)	0(0)	8 (14.6)	25 (45.5)
45 – 54	11(19.9)	6 (10.9)	1(1.8)	18 (32.7)
55 – 64	4 (7.3)	1(1.8)	1 (1.8)	6 (10.9)
≥65	0 (0)	0 (0)	0 (0)	0
**Occupation**				
Farmers	6	0 (0)	0 (0)	6 (10.9)
Small scale businesses	28	0 (0)	0 (0)	28 (50.9)
Not employed	2	0 (0)	0 (0)	2 (3.6)
Community health workers	0 (0)	3 (5.5)	0 (0)	3 (5.5)
Village Executive Officers	0 (0)	3 (5.5)	0 (0)	3 (5.5)
Hamlet Leaders	0 (0)	3 (5.5)	0 (0)	3 (5.5)
Principal Investigators	0 (0)	0 (0)	3 (5.5)	3 (5.5)
Project Managers	0 (0)	0 (0)	3 (5.5)	3 (5.5)
Field Workers	0 (0)	0 (0)	2 (3.6)	2 (3.6)
Study coordinators	0 (0)	0 (0)	2 (3.6)	2 (3.6)
**Education level**				
Did not attend school	6 (10.9)	0 (0)	0 (0)	6 (10.9)
Primary school	30 (54.6)	7 (12.7)	1 (1.8)	38 (69.1)
University 1st degree	0 (0)	2 (3.7)	2 (3.7)	4 (7.4)
University 2nd degree	0 (0)	0 (0)	5 (9.1)	5 (9.1)
University 3rd degree	0 (0)	0 (0)	2 (3.6)	2 (3.6)

### Existing Community structures engaged in IHI research

From the interviews it appears that different community structures were involved by researchers, when they were seeking entry into the community for implementation of research related activities. These structures included village government leaders, which comprised of Ward executive officers, Village chairmen, Village executive officers, Hamlet leaders and Community health workers. One of the male community respondents from Matimbwa village said that any research intended to be done in the community must pass through village office, which in turn consult community health workers and hamlet leaders:

*Every research before it is done must be reported at the village office, and after that it’s the community health worker who is supposed to do sensitization and all leaders know it’s her duty together with the hamlet leaders (CR(4),male, FGDs).*

Similar results were reported by one of the principal investigators that community structures were engaged in health research at IHI-Bagamoyo. These community structures were Village government leaders, which comprised of: Ward executive officers, Village Chairmen, Village executive officers and Hamlet leaders. On top of that, the respondent reported about engagement of community health workers, and advisory board as community structures to reach the community though had never had experience with advisory board during research related activities.

*There are different structures or levels which are involved to reach the community of which researchers must pass through. These are village government, community health workers, there is also a board which I have heard but not having experience with it known as Community Advisory Boards (PI (1), male, IDI).*

### Approaches used by community structures during engagement activities

Several approaches of engagement were reported to be used when engaging the community into research activities through community structures. Public meetings organized by community structures in which the study team members were introduced to the communities, was one of the reported approaches used. Public meetings were also used to share general information about IHI activities and specific research goals. Household visits by Hamlet leaders and CHW was another reported approach. This was specifically used for deeper conversation about a particular research at the household level where potential participants could be recruited.

*Village chairmen or village executive officers, make announcements so as to get audience, hence there after a meeting is done so that the researchers could convey their message. The hamlet leaders and community health workers (CHWs) make household visits so as to inform the people (PM (3), Male, IDI).*

The use of outpatient attendance at hospital/dispensary was also among the reported approaches used. This was so necessary when the information sharing needed to be undertaken at the health facility for studies involving patients.

*On the side of the hospital, we target patients who come at the Hospital as normal routine for treatment, but also Community health workers who are in the Villages are involved to engage the community at village dispensaries or household(SC (2), male, IDI).*

The use of Health District Coordinators was also one of the reported approach. For example, PIs of a Tuberculosis (TB) project under IHI, were reported to work with TB district coordinators to identify patients with TB to be sensitized to participate in TB research project. In this instance, sensitization meetings would be at the health facilities where patients seeking health care. In this case, the sensitization would be done by nurses, doctors or people from the research team.

*Most of the time apart from Community Advisory Boards……….as for me I do research related to Tuberculosis, so I use District Coordinators for Tuberculosis, nurses of the specific area to patients with Tuberculosis (PI (2), male, IDI).*

The use of some members of village leaders/representatives families through their both informed assent and consent as project or research participants was also reported as an approach used to facilitate community engagement into health research/projects which happened to be carried out at IHI. This was said to contribute to encouraging members of the community to participate in the research projects

*I participated as a parent, my child was involved in research and I also participated as a leader whereby I was involved in sensitization processes” (VEO (2), Male, IDIs).*

In addition, routine health care campaigns in the community to create awareness of the particular diseases such as Tuberculosis day (TB day), Malaria day and HIV day; sometimes researchers use these platforms/campaigns to facilitate engagement around specific research project:

*There are campaign such as TB, Malaria and HIV day, these platform are used to engage the community that today…. In short there are normal routine for patients who goes at the Hospital or dispensaries and awareness of the certain diseases.*

### Relationship between Community Structures and the research institute/ Team/ Researchers

A core role of engagement is to strengthening positive relations between research programmes/projects/staff and the communities hosting the research. Researchers reported that the relationship with community structures was through the support given during research activities. The community structures were described as an important link between the community and the researchers; they raised community concerns about specific research activities and promptly facilitated timely and appropriate response from the research teams. Issues raised included challenges that the study may be experiencing in the community, misunderstandings of the work of IHI, rumours, among others.

*The relationship with the Village Government is by them acting as gate keepers of the Village, Village Government give permission to talk to community service board and work in the particular Village, communication with CAB Chairman is done before talking to the Villagers and information on particular research is shared, thereafter go to Villages and deliver information and inform us in case of any rumours, CAB used to be ears for the Institute in the Community, if there are romours they tell us, they tell us if there is a problem then we go early to solve that problem (PM(1), Male, IDI).*

Furthermore, researchers reported that they have good relationship with the community in which they carryout research related activities, and that this relationship is maintained through having regular communication with the community and conducting quarterly meeting.

*Our relationship is good and most of the time when we start research project, we call them, we introduce our research project to them, telling them what we want to do, what are objectives of our research, what are their responsibilities and we give them chance of asking questions and we clarify things that are not understood, but we always have quarterly meeting so we can know the challenges, and to ask them to do more emphasis on some areas (PI (2), Male, IDI).*

Despite this, the relations between the community and research teams were described by one of the community respondent as one-directional, since it was not maintained past the study period, thus it was expressed that researchers only sought community gatekeepers and undertook engagement when they needed to undertake research activities:

*In previous years during RTS, S research project activities, engagement activities were funded by the project, after the project phased out there were no more coordinated engagement at the institute and every incoming project had their own way of engaging the community (PM(2), Female,IDI).*

### Reported Strengths of Involving Community Structures in Health Research/Projects

In the conducted interviews with the researchers, it was revealed that the advantages of the community structures were linked to the support that the researchers received at the time of initiating their research projects; for instance, communicating to researchers of any rumors or problem encountered in the community about the research, and in making sure that research is accepted in the community.

*Community structures have been doing very great job specifically during RTSS (Mal 40, Mal 50 and Mal 55) because it was a new vaccine and it had a lot of challenges, but through those structures we did not experience rejection in the Community, and if there were problems in the Community, we were able to solve regarding any romours for all the years almost 7 years. Village Government cooperated with us effectively (PM (2), Male, IDI).*

Furthermore, researchers, reported that community structures were most helpful during recruitment phase of the studies, as they helped in solving small conflicts among the community regarding a research project:

*There is big advantage like those I have said, there is simplicity in doing the work, being accepted and get participant. They have helped in getting people and the real results(PI (2), Male, IDI).*

Moreover, in some projects the community members who happened to have been participated in the project were benefiting from the project through getting free medical checkups, treatments and vaccinations:

*Malaria project has helped us a lot, in our families, personally my child had head problem and was assisted in treatment, to the point of taking my child to Muhimbili hospital (CR (23), Female, FGDs). It was Malaria project; they were testing Malaria to the children and those with other problems were treated. They told me this project is for Malaria trial to children, my child was 3 months and had the problem of blood, they took her to Muhimbili hospital for treatment (CR (12), Female, FGDs).*

Another support include increasing the community trust towards the research team, based on positive image about the study which might have contributed to researchers meeting their research goals on time. It was reported that the community structures were most helpful during recruitment phase of the studies as, they helped in solving small conflicts among the community regarding a research project:

*Village Government cooperated with us effectively (PM (2), Male, IDI).*
*There is big advantage like those I have said, there is simplicity in doing the work, being accepted and get participant. They have helped in getting people and the real results, however, disadvantages are these; although community structures work very hard, limited budget for their engagement activities is one of the reasons that we have been struggling hard to implement research, but we try a lot for these research to have small budget of Community engagement (PI (2),Male, IDI)*

### Reported weakness on community engagement activities

Different weaknesses regarding community engagement processes and activities by IHI researchers/ PIs were reported. As one of the notable weaknesses, researchers reported a progressive decline in recruitment of participants into research activities due to unsustainability of engagement plans and their implementations.

*Lack of effective engagement and transparency, that is why participants drop from the project, because they do not understand and have no proper information regarding the research project (CR (8), Female, FGD).*

Moreover, researchers reported being nervous on sustainable engagement of community structures into research related activities due to inadequate budgets for engagement activities.

*Disadvantages are these community structures work very hard but when it comes on the issue of Community engagement and miss budget for that we will fail and struggle to implement research, but we try a lot for these research to have small budget of Community engagement (PI (2), Male, IDI).*

In addition, little engagement about the research after enrolment, limited awareness of the study objectives, lack of proper feedback of results/findings, mobility (people moving to another region), and false promises associated with payment of community members who assist studies in the community were the reported weaknesses:

*Many researchers go outside the agreement, for example they put a trapping material for trapping mosquitoes and say it will stay for one month and it goes up to two months, a person is being told to remove the trap at twelve in the morning and twelve in the evening and is not paid as agreed. At the end of the day will not work effectively (VEO (4), Female, IDI).*
*It is good for every project to think of community engagement, enough budget should be put aside, for example budget set of going to Kiwangwa, but that person in Kiwangwa what will she/he use in moving around to look for participants, resources should be open and allocated to enable activities of community structure (P(3), Female, IDI).*
*They may not get good results because of the challenges, where they see there is a need of doing research they should do, but remember to give us feedback in public meeting, it helps in educating the community (VEO (5), Male, IDI).*

Furthermore, community members reported more other weaknesses associated with involvement of the community structures during engagement activities, and how to improve them. They suggested that processes of informing the community can be improved to a point of using media such as radio, television, posting announcement so that people can be aware of what is going on around the community.

*Other information regarding research should be given through media, posting announcement. But going through community structures is the proper way because they have to know any project that comes in the village” ( CR (12), male, FGD).*

Also it was reported that researchers should find time to visit areas where community structures have been sharing information on research activities. These will therefore, emphasize important elements of the information, and to clarify some of the areas that are difficult for community members to understand.

*There should be close communication between CAB members, village government and researchers. Researchers should frequently visit the community to emphasis on what have been said by the community structures. There may be discrepancies on information shared in more than one village (PM (1), male, IDI).*

Researchers also reported that it is important to develop a list of frequently asked questions, and find a way of simplifying scientific words in a language that can be understood by people of all levels. This will help in elaborating the different aspects related to research activities.

*Develop list frequently asked questions and create answers and be given to those who are very close to the community. For those which have no answers, they should make reference from researchers” (PM (1), male, IDI)*
*On my views there should be close communication, those CAB members, Village Government, researchers should be going often so as to put emphasis on what have been said, there may be difficult information that have been shared differently in more than one village. To generate frequently asked questions and to create answers and be given to those who are very close to the community. For those which have no answers they should make reference from researchers (PM (1), Male, IDI.).*

Researchers also recommended strengthening the department of community engagement at all branches of IHI, by establishing reliable and strong community advisory boards. Resources should be allocated in this department for it to work effectively. In addition, there should be good relationship between researchers and the communities whereby researchers can contribute in community activities like building of the dispensaries, schools and IHI doctors can also work with district hospitals and help in providing treatment services. This is one way of maintaining regular contact with the communities.

*Sustainable way, I think is to build strong CAB which will strengthen department of community engagement in all IHI branches. There are still some gaps, I think because resources have not been allocated, these will help to build relationship and establish engagement with the community ( PI (1), male, IDI).*
*It is good to have regular contact with them, if there is anything needs to be doing in the community so as to collaborate with them sustainably, building of dispensary, schools et c will strengthen good relationship, for example IHI doctors can collaborate and involve in treatment activities at the District Hospital, because people are the same and even if we go they recognize us, it help us to be known as Institute to the community. ( PI (3), Female, IDI).*

They also suggested that there should be adequate funds in their budgets allocated to helping CHW with their mobility in the villages during participant recruitment phase. This has been missing in their budgets which make this activity in engagement difficult to implement:

*It is good for every project to think of community engagement, enough budget should be put aside, for example budget set of going to Kiwangwa, but that person in Kiwangwa what will she/he use in moving around to look for participants, resources should be open and allocated to enable activities of community structure (P(3), Female, IDI).*

## Discussion

### Potentiality of involving community structures into health related researches/projects at IHI

Drawing onto the findings, and based on community engagement through community structures as observed in this study, a number of issues emerge: At IHI, different forms of community structures are often engaged in health research but the common one includes community-recognized leaders/gatekeepers/representatives. These leaders are important in gaining permission to the community for a research to be conducted in an area. The growing evidence also suggests that community structures often act as bridge between the community members and research team, and that this role is important in strengthening research activities in a community, in promoting social-cultural consideration of communities in research activities, in promoting inclusion of community voices in research activities, and can contribute to meeting recruitment and retention targets within time ^2, 36, 37^. The evidence also suggests that community structures can wield considerable power gained in their intermediary role. Such power can be welded positively to promote mutually beneficial conduct of research in the community, and it can also be welded negatively through controlling the information (and promoting misinformation) that gets to their social network especially if the demands of the community structures are not met ^38^.

Based on the reported findings, it’s obvious that community structures are the key to success of any research projects happened to have been conducted at IHI-Bagamoyo Centre. This is the case as it appears that the members of community were more willing to participate in any research related activities when approached for engagement by their village government leaders, community advisory board members and health leaders rather than by researchers themselves. Furthermore, it is clear that; trust, sense of being respected and assured safety of participation and after, and gained confidence are the key drivers for getting community to participate in research activities. However, the acquiring of these drivers by the participants would not be possible without the presence of community structures between the researchers and community. Several studies regardless of their designs have as well highlighted this observation. For instance, it has been reported that community members before could be recruited for participation in any research project need to trust the researcher/research teams and being assured of their dignity and safety during and after participation ^27, 39^. This observation has also been supported by Dada *et al*. that interdisciplinary community liaison and social science teams, which under the present study are referred to as community structures, when work with a clinical team/ researcher/research team can strengthen trust within member of community and thus, increase participation of members of community into research related activities ^40^. Another study by Dutta *et al*. also stressed the importance of community leaders in building the trust to the community members so as to get them participating in research activities ^41^.

### Approaches used in facilitating community engagement at IHI

From the findings it is clear that successful engagement of the community members into the health research/projects is due to the approaches used. The used approaches facilitated the spread of information about the existence of any proposed projects/research activity to be done within the area. Through the use of public meetings and household visits, trust and confidence were built among the community about the new research/ projects to be carried out in their area. This in turn increased the turnout of the community members to participate into the research/health activities. Using the community structures in organizing public meetings have also been reported by other scholars as one of the successful approach to get community member to build trust and confidence to participate into health research/project activities ^38, 42^. In addition, the use of some members of village leaders/representatives families as research participants cause other community members to build trust over the running project/research activities and thus get willing to participate ^43^. The use of outpatient attendance at hospital/dispensary and Health District Coordinators minimizes unnecessary reactions from the participants as people involved in the engagement which are doctors, nurses and community health workers are the well known to all members of the community. Besides, community members before can decide to consent for participation are likely to consult them for advice or to alleviate their fears about possible adverse outcomes of the intervention ^44^. These approaches are less costiful and save time.

### Strengths and improvement of community engagement in the health research

Different advantages associated with engagement processes were reported. These were assurance of the safety of the community members as regard per research projects as this help members of the community to trust researchers and the activities associated with research. The community in general also benefit from community engagement by being protected from unethical health research/projects. For instance, being community gatekeepers, permission to get entry into the community is at first supposed to be sought from community structures, and this was said to be important in gaining entry to the community and in reassuring communities of the goodwill of the researchers ^6, 45^. However, the improvement of the engagement processes were suggested by the community members, which are additional to the previously used method of conducting general public meeting of which most of the community members hardly attended; and the few that attended those meetings, found it difficult to grasp all the information regarding a certain research. Additional complementary engagement activities suggested include visits at household of the community members to meet head of the house and other family members, use of radios, television, posting announcement at places where people tend to gather for different purposes like in the market places, football play grounds, bars and restaurants. As reported elsewhere ^46^, additional methods are likely to emphasize what have been communicated by the other method, and will help community members to understand more when different method are used to inform them of the certain research project to be initiated in that area.

The findings also described the importance of simplifying difficult scientific words in a way that makes it easy for community to understand. This goes along with the compilation of questions and answers for the frequently asked questions to ease the communication with the community structures during the engagement activities. This suggestion emanated from the observation that community structures often failed to communicate same message to several villages without distorting the meaning ^38^. This can contribute to miscommunication of the particular research purpose. This is because often health research involves consideration of information, unfamiliar process and terminologies that require repeated sessions of information-giving. In addition, and to support good engagement processes, it was suggested that adequate funds in a budget should be allocated to strengthen the community engagement department. The ethical importance of community engagement is underpinned by its value in identifying socially and culturally appropriate consent processes and ethical conduct of different types of studies ^7, 47– 50^. Therefore, having a well-established community engagement system will help researchers know what the needs of the community are, and how best these can be addressed, and create relations of mutual trust and respect between researchers, community and community structure ^50^.

### Weakness observed during community engagement

Some weaknesses as far as the conduct of community engagement is concerned in the study areas were observed. One of the major weaknesses is about the roles played by the community advisory board (CAB) in bringing close the researchers and the community. From interviews, it appears that, the established community advisory board is not doing its role effectively to the extent that both researchers and community members call for its interventions in facilitating information flow between the researchers and the community. Since from its establishment, it was expected to be at forefront in building successful collaborations between the researchers and the community. On contrary, village government leaders and district health coordinators but not community advisory board, were the key active community structures reported in this study. Worse enough some community members reported of being unaware of what the advisory board is doing, hence recommended some roles that the board should adopt so that to easy communication flow from researchers to the community members. Contrary to the observation of this study, Shubis *et al*. reported that, the said CAB has led to improved relations with the community and facilitated the recruitment of study subjects in Bagamoyo district ^29^. The observation of the current study could be attributed to budge constraints as was reported in the result sections. Furthermore, for building strong and efficient CAB, in terms of carrying its responsibilities effectively, Mlambo *et al*. conclude that; clearly defining the scope of the CAB, addressing issues of CAB independence, the CAB budget, providing emotional support for CAB members, and providing continuous training and capacity buildings are the key aspects to be considered ^51^.

Another observed weakness over community engagement process is regarding the establishment of sustained conducive working environments between the community structures and the researchers. Since it is apparent that community structures must be in place for the research activities within the community to be accepted and supported ^52– 54^, then their working environments should be given priority. Little or no budgets for their activities were reported in this study. This is serious weakness that needs immediate attention by the IHI centre. This weakness has mainly been contributed by poor coordination between the community advisory board and the researchers. It was expected that the IHI could allocate the budget for the community structures activities through the community advisory board rather than depending directly from individual research projects which are of short term goals ^29^. Seeking community participation not only is done in health research but also in provision of other health services that demand community participation for their success. In one study aimed at exploring factors that hinder Community Participation in Developing and Implementing Comprehensive Council Health Plan in Manyoni-Tanzania, reported that lack of awareness of the program by the community stakeholders, poor communication and information sharing between local government leaders/committees, unstipulated roles and responsibilities of board related organ, and lack of financial resources for implementing local government structures activities were the main identified factors ^55^. Another study reported that economic issues among the health care committees, whose responsibility among others is to get community members to participate in health projects, were hindering them from performing their duties effectively ^56^. 

### Limitation of the study

Our research findings are not without limitations. First, we were not able to capture data in urban community due to time and budget constraints. In addition, the voices of the young people and naïve group in the villages where this research project was conducted are missing, but also the voices from the urban areas of those that participated and not participated in IHI research activities for the past 5 years, were not represented. Furthermore, the research was not able to capture data of the community members who have never participated in IHI research due to time constraints. Therefore, perceptions and experiences from these groups on the systematic functioning of the community structures, were not captured so as to have comparable data from rural and urban areas. The data would have helped to have a wide understanding of the perceptions and experiences towards community structures that have been facilitating engagement in health research

## Conclusion

This study was conducted to understand the systematic functioning of the existing community structures in facilitating engagement in health research. The community structures involved in the engagement seemed to have been influenced by the type of research project and kind of participants needed. Weaknesses identified included inconsistence research feedback, insufficient engagement for participants to capture study project objectives and false promises by researchers to community stakeholders. Therefore, there is a need to strengthen community engagement system at IHI which will coordinate research activities so as to address the reported challenges. Proposed ways of strengthening engagement with the community and researchers included use of various media of engagement such as radio, television, posters and regular meetings with the community during the course of a study. Subsequently, this will enhance understanding of the research information by the community members and community structures, as well as help raise some of the issues that could be considered when developing a research protocol. Frequent interactions will also enhance information sharing with the targeted population. This can be done by developing a list of frequently asked questions and answers so to facilitate the understanding of the community stakeholders

## List of abbreviations and symbols

BRTC      -      Bagamoyo research and training center

CAB        -      Community advisory board

CDC        -      Center for disease control

CERC      -      Community engagement research core

CES         -      Community engagement studio

FGD        -      Focus group discussion

HIV         -      Human Immunodeficiency syndrome

IDI           -      In-depth interview

IHI           -     Ifakara Health Institute

IRB          -       Institution review board

CHW        -    Community health workers

KEMRI   -     Kenya medical research Institute

DED       -         District executive director

NIMR       -       National Institutes of Medical Research

PI   -              Principal Investigator

PM   -           Project Manager

SC   -            Study Coordinator

FW   -           Field worker

HL   -           Hamlet leader

CR   -           Community respondent

VEO   -        Village executive officer

## Data availability

### Underlying data

According to the Institutional Review Board (IRB) of the Ifakara Health Institute, we are not allowed to make the transcript data from the focus group discussions or the in-depth interviews publicly available due to fact that policy for data circulation has to be signed. Quotes from the transcripts are provided in the text as intermediary data. Interested researchers should contact the corresponding author or irb-submission@ihi.or.tz. Data access will be granted under the following conditions: (i) signing data sharing agreement; (ii) waiver of the informed consents by IRB if the justifications are considered to be ethically and scientifically sound, since the consent forms clearly stated that the participants’ data will not be shared outside the Institute.

### Extended data

Figshare: Community-networks that facilitate engagement in health research: Ifakara Health Research Institute-Bagamoyo case study, https://doi.org/10.6084/m9.figshare.14113229.v1 ^13^.

This project contains the following extended data:

- Community networks_Community stakeholders interview guide,
- Community networks_Researchers interview guide
- Community_networks Group discussion guide.up

Data are available under the terms of the Creative Commons Zero “No rights reserved” data waiver (CC0 1.0 Public domain dedication).
