# Supplementary material for: Community-structures that facilitate engagement in health research: Ifakara Health Research Institute-Bagamoyo case study
Source: AAS Open Res. 2022 Mar 23;4:13. Originally published 2021 Mar 11. [Version 2] doi: 10.12688/aasopenres.13187.2 (PMC11303950; doi:10.12688/aasopenres.13187.2)
Supplement: Peer review history

# Reviewer report: Reviewer response for version 2

**DOI:** 10.21956/aasopenres.14476.r31380

**Published:** 2024-08-06

**Authors:** Gillian Frances Black

**Affiliations:** 1 Sustainable Livelihoods Foundation, Wynberg, South Africa

This paper presents the findings of research undertaken to explore

 1.  - the nature of existing community structures that are or could be engaged in health research at the Ifakara Health Institute (IHI), Bagamoya, Tanzania

 2. - the approaches used by IHI research center to engage the community into health research and project activities

  3. - the strength and weakness of working with identified community structures

  4. - the nature of existing relations among IHI research center research teams and the community structures

 The study population was categorized into three groups: i. ‘ordinary’ community members, ii. community leaders/ representatives and iii. researchers including research coordinators, research managers, field workers and principal investigators at the IHI-Bagamoyo center.

 The research methods comprised six focus group discussions (FDGs) involving 36 community members, nine in-depth interviews (IDI) with community leaders/representatives and 10 IDIs with researchers at different levels.  

 The authors use inductive and deductive approaches in analysing the data. Findings were analyzed by comparing responses across different groups in relation to their experiences and considering verbatim quotes.

 I have reviewed this article as a third reviewer following the authors' responses to comments made by reviewers 1 and 2. 

 Overall, the research study focused on an important and timely topic and has the potential to meaningfully inform the Ifakara Research Institute in particular about how it can strengthen and improve its community engagement efforts.   

**Is the work clearly and accurately presented and does it cite the current literature?**

Partly. The manuscript will benefit from being thoroughly proofread for edits to the language and grammar throughout. In making this comment I recognize and respectfully appreciate that English may not be the first language of the principal writer. As a part of this, there are some very long sentences that can be split up. There are also some spelling typos and areas of repetition to be addressed.

 In the Introduction, sub-section ‘The Ifakara health institute-Bagamoyo’ the authors give abbreviations RTS and S, in reference to clinical trials. The names of these interventions need to be given in full, please.

 In the last paragraph of the Introduction (Therefore this study was designed to explore:…) the order of the four analytical themes presented is not quite the same as the order of themes presented in the Findings. Please remedy for consistency.

 Also, please include a methods citation for inductive and deductive approaches to data analysis.

**Is the study design appropriate and is the work technically sound?**

 Yes, it appears so. There are however some methodological aspects that need to me clarified (see below).

**Are sufficient details of methods and analysis provided to allow replication by others?**

No.

 In the Methods section under ‘Tools development and data collection’, please indicate if all of the in-depth interviews were done with individuals, or if any of these types of interviews were conducted with more than one respondent at a time.

 In the section on Data management and analysis:

 1. The authors state that an open coding framework was developed by reading the transcripts, codes were grouped into analytical themes and a framework matrix was developed based on these themes. What format were the transcripts in? E.g. were they digital, perhaps entered into Microsoft Word, or some other type of electronic document? It is also important to clarify whether the analysis process was done entirely manually (i.e. without the aid of any data analysis software) or if software of any type was used. The authors could provide readers with a deeper understanding of the analysis process by showing the framework matrix as a Table in the paper.

 2. The authors state that ‘Findings were analyzed by comparing responses across different groups in relation to their experiences and verbatim quotes’. It is not clear whether the data was examined for significant or important differences in the way that the 3 core respondent groups replied to the thematic questions. Please can the authors include a sentence or two to clarify if the data was stratified per respondent group prior to analysis, or if all the data was considered as a single data set.

 More learning could have been gained through this study regarding differences in perspectives observed both between and within the respondent groups. 

 Please correct this statistic. ‘This study was conducted in Bagamoyo district, Pwani region, which has a population of 31,1740…’

 The first subsection of Findings ends with the following sentence: ‘Based on occupation and education level majority of respondents were in small scale businesses class (Entrepreneurs 50.9% (n=28)) and possessed primary education (69.1% (n=38)) as the highest level of education’. The authors need to please clarify which respondent group or groups these statistics are referring to.

 The second subsection of Findings (Existing Community structures engaged in IHI research)  begins with the sentence: ‘From the interviews it appears that different community structures were involved by researchers, when they were seeking entry into the community for implementation of research related activities’. Please can the authors clarify the use of the word ‘interviews’ here: does this refer to the focus group discussions done with the ordinary community members AND the in-depth interviews with the other two respondent groups?

 Also in Findings, in the section on ‘Reported Strengths of Involving Community Structures in Health Research/Projects’, we find the following text:

 ‘Moreover, in some projects the community members who happened to have been participated in the project were benefiting from the project through getting free medical checkups, treatments and vaccinations: *Malaria project has helped us a lot, in our families, personally my child had head problem and was assisted in treatment, to the point of taking my child to Muhimbili hospital (CR (23), Female, FGDs). It was Malaria project; they were testing Malaria to the children and those with other problems were treated. They told me this project is for Malaria trial to children, my child was 3 months and had the problem of blood, they took her to Muhimbili hospital for treatment (CR (12), Female, FGDs).*

 The link between the above-reported finding and strengths of involving community structures needs to be clarified.  

**Are the Conclusions drawn adequately supported by the results?**

Partly.

 Linked to my initial comment, the Discussion can be tightened up through review by the authors for English language and repetition.

 In the Discussion sub-section ‘Approaches used in facilitating community engagement at IHI’ I wasn’t sure what is meant by ‘unnecessary reactions from the participants’. ?

 Whereas the Conclusions draw upon the reported weakness of community engagement that were identified through the study, the conclusions also need to also draw upon the reported strengths of involving community structures in health research. The identified strengths are also important for informing the recommendations that are presented in the Conclusions. Furthermore, the Conclusions section can be bolstered by a statement about how the key findings of the study, and the resulting recommendations, could be helpful to other research institutions in Tanzania, and in the African region, beyond IHI.

Is the work clearly and accurately presented and does it cite the current literature?

Partly

If applicable, is the statistical analysis and its interpretation appropriate?

Not applicable

Are all the source data underlying the results available to ensure full reproducibility?

Yes

Is the study design appropriate and is the work technically sound?

Yes

Are the conclusions drawn adequately supported by the results?

Partly

Are sufficient details of methods and analysis provided to allow replication by others?

No

Reviewer Expertise:

Community engagement and involvement in global health research

I confirm that I have read this submission and believe that I have an appropriate level of expertise to confirm that it is of an acceptable scientific standard, however I have significant reservations, as outlined above.

# Reviewer report: Reviewer response for version 2

**DOI:** 10.21956/aasopenres.14476.r29187

**Published:** 2022-04-07

**Authors:** Shelley Lees

**Affiliations:** 1 Department of Global Health and Development, London School of Hygiene and Tropical Medicine, London, UK

The authors have addressed the reviewer's comments and the paper is now much improved. This is now a very well written paper that contributes to the growing academic field of community engagement.

Is the work clearly and accurately presented and does it cite the current literature?

No

If applicable, is the statistical analysis and its interpretation appropriate?

Not applicable

Are all the source data underlying the results available to ensure full reproducibility?

Yes

Is the study design appropriate and is the work technically sound?

Yes

Are the conclusions drawn adequately supported by the results?

Partly

Are sufficient details of methods and analysis provided to allow replication by others?

Partly

Reviewer Expertise:

Anthropology

I confirm that I have read this submission and believe that I have an appropriate level of expertise to confirm that it is of an acceptable scientific standard.

# Reviewer report: Reviewer response for version 1

**DOI:** 10.21956/aasopenres.14296.r28466

**Published:** 2021-04-28

**Authors:** Shelley Lees

**Affiliations:** 1 Department of Global Health and Development, London School of Hygiene and Tropical Medicine, London, UK

This is an interesting paper but does need some improvements.

**Introduction:**

1. I think you need to decide whether you are talking about networks or structures or both. The use of both together is a bit confusing.
2. The second paragraph under Roles of Community Networks/Structures needs some editing. Especially the section starting ‘Engagement with the community for any successful…’ It is quite a muddled few sentences.
3. I am not sure from an ethics perspective whether the community should feel part of the research. Surely only the participants are. Can you clarify what you mean by this?
4. I am not sure why you have provided an example of community engagement from the US. I would suggest providing other examples from Africa. You could look at these papers, but there are others.

- 'How informed is consent in vulnerable populations? Experience using a continuous consent process during the MDP301 vaginal microbicide trial in Mwanza, Tanzania' by Vallely *et al. *(2010 ^1^).
- 'A model for community representation and participation in HIV prevention trials among women who engage in transactional sex in Africa' by Shagi *et al.* (2008 ^2^).
- 'How can community engagement in health research be strengthened for infectious disease outbreaks in Sub-Saharan Africa? A scoping review of the literature.' by Vanderslott *et al.* (2021 ^3^).

**Methods:**

1. It is unclear how many participants were involved. Under study respondents it says 55. But under Data Collection it says 6 FGDs and 49 IDIs. Surely that is more than 55.

**Findings:**

1. Whilst the community network was described in the findings, I found it hard to visualise the different networks. Could a diagram be provided?
2. The section ‘Approaches used by community structures/networks’ is very short and only two approaches are provided. And the second quote does not seem to be about community engagement, rather help-seeking.
3. Again the section on ‘Advantages of involving community networks/structures’ is very short and only seems to elaborate advantages to research teams. What are the advantages to the community?
4. Overall the findings are very thin, given the number of people involved in the research. There seems to be very little nuanced analysis of the experiences from the community perspective.

**Discussion:**

1. The discussion present results that are not in the findings section. For example the sentence ‘The results of this study also suggest that the respondents felt that relations between researchers and community structures are skewed towards the researchers’ interests and needs’. This is an important finding and needs to be presented in the findings.
2. Another point in the discussion about improving engagement processes is not in the findings. Or the observation that community structures often failed to communicate the same message.
3. The discussion would be strengthened by reference to other literature. The reference list is very short and a lot has been written by other authors that would be helpful to compare these findings to.

**Conclusion:**

1. This is currently a summary of the discussion and needs to be a more nuanced conclusion about the research.

Overall there is a need to edit and check grammar.

Is the work clearly and accurately presented and does it cite the current literature?

No

If applicable, is the statistical analysis and its interpretation appropriate?

Not applicable

Are all the source data underlying the results available to ensure full reproducibility?

Yes

Is the study design appropriate and is the work technically sound?

Yes

Are the conclusions drawn adequately supported by the results?

Partly

Are sufficient details of methods and analysis provided to allow replication by others?

Partly

Reviewer Expertise:

Anthropology

I confirm that I have read this submission and believe that I have an appropriate level of expertise to confirm that it is of an acceptable scientific standard, however I have significant reservations, as outlined above.

## References

[1] : How informed is consent in vulnerable populations? Experience using a continuous consent process during the MDP301 vaginal microbicide trial in Mwanza, Tanzania. *BMC Med Ethics* .2010;11: 10.1186/1472-6939-11-10 10 10.1186/1472-6939-11-10 20540803 PMC2893460

[2] : A model for community representation and participation in HIV prevention trials among women who engage in transactional sex in Africa. *AIDS Care* .2008;20(9) : 10.1080/09540120701842803 1039-49 10.1080/09540120701842803 18825513

[3] : How can community engagement in health research be strengthened for infectious disease outbreaks in Sub-Saharan Africa? A scoping review of the literature. *BMC Public Health* .2021;21(1) : 10.1186/s12889-021-10348-0 633 10.1186/s12889-021-10348-0 33794820 PMC8012744

# Reviewer report: Reviewer response for version 1

**DOI:** 10.21956/aasopenres.14296.r28495

**Published:** 2021-04-28

**Authors:** Nicola Desmond

**Affiliations:** 1 Department of International Public Health, Liverpool School of Tropical Medicine, Liverpool, UK

This is an important and relevant paper that sets the scene for the development of a comprehensive and 'inclusive' process for ongoing and sustainable community engagement approaches within the Ifakara Health Research Institute in Bagamoyo, Tanzania.

 The paper begins with a background on community structures in place and how functional community networks are in providing pathways for information to flow amongst participants in research. The paper uses a qualitative descriptive approach which is appropriate for the research objectives and presents the results descriptively using quotes from participants. The discussion leads neatly to the conclusion that a broader engagement approach is needed that moves beyond specific project-defined needs and purposive sensitisation approaches targeting particular research. Both the purpose and the conclusions of the paper are clear and contribute to the growing literature recognising the current gap in true community engagement that may impact negatively (either immediately and linked to the research recruitment and retention or in the long-term impacting on wider community trust and rumours surrounding research). I have answered partly to some of the questions above and will explain this below:

1. I wasn't sure whether the paper has the appropriate title since it defines research on community networks that facilitate engagement in health research but I am not convinced the paper is actually about networks but rather about community-based leadership structures used by research to promote the sharing of information about research. I am not convinced that the research conducted for this study explored the community networks themselves. With the current Tiel I would have expected more research/exploration/identification of existent networks within communities and their relative functionality for creating/sustaining community trust around research. If this research did not explore that then I think change the title to community structures rather than networks.  
2. An issue that needs to be considered, perhaps in the discussion is about the purpose and aims of a CE approach - this has been done to an extent but more consideration could be given to the purpose of engagement as a route to creating dialogue, ownership, mutual benefits, accountability etc. Is the 'voice' of the community tokenistic and ethically defined by the needs of the research or is it participatory and epically driven?
3. There doesn't seem to be any critical consideration of the role of the CAB and its 'representativeness' of the community - one quote highlights the lack of communication between community members and CABs (p8) but this is not really explored or discussed later in the paper - one of the key drivers for the WT approach to engagement was early recognition that tokenistic approaches may not truly represent communities and this needs more careful thought in this particular setting.
4. I suggest including a bit more detail of reflexivity./researcher positionally in the methods section particularly describing the RAs and lead researcher and their relationships with the community since this intrinsically informs the responses they receive in collecting the data through interviews/FGDs. An example would be to describe whether they are insiders/outsiders - do they come from the villages or are they from elsewhere? Given the emphasis on the socio-cultural dimensions of research responses and the over 120 ethnic groups in Tanzania this might be important to understand, particularly since coastal Tanzania has specific socio-cultural norms that often differ from those elsewhere in the country. 
5. There is a slight disconnect between the results and the discussion. It comes across as though the paper is driven by the objective of arguing for a funded engagement programme (something I would definitely support) but the results don't necessarily lead to this. Other things mentioned in the discussion section such as the distortion of meanings as messages get relayed in communities is not presented in the results.

The paper also needs a good proof read to improve some of the sections where the English is not quite there.

 Finally I agree with the purpose and aim of this paper but would like to see some broader interpretation and implications beyond the specific setting. The recommendation to move from a research needs driven focus to a community driven focus in engagement approaches is something that could be considered beyond the argument for funding an engagement programme in this setting.  

 To conclude this is important work that will contribute hopefully to an agenda that supports the development of an engagement process at the Ifakara Institute that moves beyond research specific needs and aims but as a paper published internationally more consideration of the broader issues from a normative perspective of engagement rationales might be more relevant to the audience. This can easily be done since the authors are engaged in global networks to critically contribute to improved engagement.

Is the work clearly and accurately presented and does it cite the current literature?

Yes

If applicable, is the statistical analysis and its interpretation appropriate?

Not applicable

Are all the source data underlying the results available to ensure full reproducibility?

Yes

Is the study design appropriate and is the work technically sound?

Partly

Are the conclusions drawn adequately supported by the results?

Partly

Are sufficient details of methods and analysis provided to allow replication by others?

Partly

Reviewer Expertise:

Community engagement; risk; medical anthropology; acceptability; interventions; public health; social science; qualitative research; bioethics; sexual and reproductive health; vaccines; self-care

I confirm that I have read this submission and believe that I have an appropriate level of expertise to confirm that it is of an acceptable scientific standard, however I have significant reservations, as outlined above.
